# Nanostructured SnO_2_–ZnO composite gas sensors for selective detection of carbon monoxide

**DOI:** 10.3762/bjnano.7.195

**Published:** 2016-12-22

**Authors:** Paul Chesler, Cristian Hornoiu, Susana Mihaiu, Cristina Vladut, Jose Maria Calderon Moreno, Mihai Anastasescu, Carmen Moldovan, Bogdan Firtat, Costin Brasoveanu, George Muscalu, Ion Stan, Mariuca Gartner

**Affiliations:** 1 Ilie Murgulescu Institute of Physical Chemistry of the Romanian Academy, Splaiul Independentei 202, 060021 Bucharest, Romania; 2 National Institute for Research and Development in Micro-technologies, 077190 Bucharest, Romania,; 3 Romelgen SRL, Bucharest, Romania

**Keywords:** composite thin film sensor, dip coating, low detection limit, miniaturized alumina transducer, selective CO detection, sol–gel

## Abstract

A series of SnO_2_–ZnO composite nanostructured (thin) films with different amounts of SnO_2_ (from 0 to 50 wt %) was prepared and deposited on a miniaturized porous alumina transducer using the sol–gel and dip coating method. The transducer, developed by our research group, contains Au interdigital electrodes on one side and a Pt heater on the other side. The sensing films were characterized using SEM and AFM techniques. Highly toxic and flammable gases (CO, CO_2_, CH_4_, and C_3_H_8_) were tested under lab conditions (carrier gas was dry air) using a special gas sensing cell developed by our research group. The gas concentrations varied between 5 and 2000 ppm and the optimum working temperatures were in the range of 210–300 °C. It was found that the sensing performance was influenced by the amount of oxide components present in the composite material. Improved sensing performance was achieved for the ZnO (98 wt %)–SnO_2_ (2 wt %) composite as compared to the sensors containing only the pristine oxides. The sensor response, cross-response and recovery characteristics of the analyzed materials are reported. The high sensitivity (*R*_S_ = 1.21) to low amounts of CO (5 ppm) was reported for the sensor containing a composite sensitive film with ZnO (98 wt %)–SnO_2_ (2 wt %). This sensor response to CO was five times higher as compared to its response to CO_2_, CH_4_, and C_3_H_8_, thus the sensor is considered to be selective for CO under these test conditions.

## Introduction

For the past decade, the concept of integration has been the main drive that supported research and development in the domain of smart devices for remote sensing, that is, sensors based on changes in electrical conductivity [1]. The main advantages presented by this category of gas sensors are low cost compared to other sensing technologies, low power requirements, quick response times (seconds), high sensitivity to small concentrations of a specific gas, complete sensor recovery, lightweight, and long term stability. More recently, sensors were fabricated with reduced dimensions (miniaturization or microelectronic processing) [2–3].

The oxide-based semiconductor sensors have been used worldwide to detect toxic, hazardous and combustible gases (e.g., C_2_H_5_OH, H_2_S, H_2_, various hydrocarbons, volatile organic compounds) for the safety of humans and for a better control over the surrounding environment [1–3]. Thin layers of metal oxide semiconductors were used for the first time as sensing materials in 1962, when a report was released by Siyama et al. [4] regarding a thin film of ZnO.

The principle of sensing, when a resistive chemical sensor is used, involves measuring the changes in electrical resistance that occur in the sensitive layer during sensor exposure to a certain gas, in a given gaseous environment. Under environmental conditions (measurements are performed in air) the surface of a metal oxide sensing material adsorbs oxygen as different species: O_2_^−^, O^−^ or O^2−^ (depending upon temperature).

When the target gas reacts with the adsorbed oxygen species on the surface of the sensing material, a significant change of resistance can be detected by a calibrated electronic circuit (or a more complex device) capable of “translating” the electrical signal into another type of information, like the concentration of the test gas [3]. This may be regarded as a simplified view of the sensing mechanism for this type of gas sensor.

As an example, for typical n-type semiconductors, the overall sensor resistance increases after exposure to oxidizing gases or decreases after the sensor is exposed to reducing gases. For p-type oxide semiconductors [5], the observed electrical changes upon exposure to oxidizing or reducing gases are reversed in comparison with n-type semiconductors [1].

As previously mentioned, metal oxide based sensors offer many advantages as sensing materials. The similar response to a wide range of gases is the major drawback when using this type of material, meaning that these sensors are nonselective towards a specific gas. False positives are often generated in this way. Gas selectivity can be achieved using different “sensor tuning” techniques. These include controlling the sensing temperature [6–7], the addition of a noble metal [8] or oxide catalysts [9], surface modification [10–11], manipulation of the nanostructure [12], the use of multicomponent (or composite) sensing films [13–22], electronic noses, advanced signal processing techniques (chemometrics) and advanced sensor operation techniques, such as temperature modulation [1].

In the past few years the use of composite sensors has yielded a lot of published work in the gas sensing domain [1,3,5,7–8,13–33]. However, the addition of another oxide component described in these papers involves complicated and expensive vapor preparation techniques (e.g., chemical vapor deposition (CVD) or physical vapor deposition (PVD), ion-beam or laser-assisted techniques, spray pyrolysis), expensive dedicated equipment (e.g., screen printing) or complicated wet preparation routes that require the use of special solvents and templates [23]. Usually the sol–gel technique combined in our case with dip coating is a low temperature process; it requires less energy consumption and causes less pollution – all features that make it an ecologically friendly preparation technique.

Carbon monoxide (CO) is one of the most dangerous gases present in our surrounding environment. As it does not have a specific smell, color or taste, CO detection is impossible without special warning systems [24]. When organic matter is oxidized the usual obtained products are CO_2_ and H_2_O (when complete oxidation occurs). In the case of insufficient oxygen, a partial oxidation of the organic compound occurs, with CO resulting as the main product [24]. CO is present in high concentration in underdeveloped areas or regions where fossil fuel technology is still intensely used, or in crowded cities with high levels of pollution produced by internal combustion engines. Existing commercial sensors are used as a warning system (in general, acoustic alarms are set off) to the otherwise undetectable carbon monoxide [25]. The presence of CO in very small concentrations (even at 35 ppm) in the atmospheric environment produces visible effects in human beings, such as dizziness. The intensity of the manifested symptoms increases with increasing CO concentration (at 200 ppm disorientation and loss of judgment occurs, at 800 ppm convulsions begin). The effect of CO is lethal if the concentration is higher than 1600 ppm [26].

SnO_2_–ZnO composite metal oxide sensitive films were previously obtained with good sensing results [3,23,27]. However, the previously published reports indicated that the films were prepared using expensive techniques or the obtained sensors were either nonsensitive or nonselective to CO.

The present work emphasizes that selective detection of CO in trace amounts (5 ppm) is obtained using a nanostructured metal oxide composite sensor, containing SnO_2_ and ZnO in different ratios. The sensitive layer was obtained/deposited using the sol–gel/dip coating method. The sensor cross-response was tested with other hazardous gases, namely methane (CH_4_), carbon dioxide (CO_2_) and propane (C_3_H_8_); good selectivity was found towards CO over CH_4_, C_3_H_8_ and CO_2_, so the sensor may be proposed for further development towards indoor applications in areas where gas burners are still used.

## Experimental

### Sensor fabrication

The miniaturized alumina transducer was designed and developed using specific microtechnology processes, clean room facilities and software. The transducer fabrication involved two major steps: a) simulation for choosing the optimum design of the transducer’s Pt heater, and b) fabrication of the transducer using mask technology and laser lithography on dedicated equipment, namely the Heidelberg DWL66FS, and wafer processing using photolithographic process with double-side alignment, metal etching and lift-off process.

### Simulation

The Pt heater was simulated in order to optimize the design for uniform heating of the sensor active area and minimizing the power consumption. The COMSOL Multiphysics^®^ finite element analysis (FEA) tool was used for effective modelling/simulation of the heater components for the transducer. The analyses allowed for a quick and effective heater optimization directly from the design stage (without effective fabrication of the transducer). The simulations were performed using a coupled electro-thermal interface that considered both the Joule heating of the resistor and the heat convection in the surrounding environment.

The microheater design was optimized by means of these analyses with the purpose to generate high temperatures in the electrode region with as low as possible input voltage. Different shapes (serial and parallel resistors) and widths (from 350 to 1200 μm) were simulated for the Pt heater of the transducer.

**Figure 1:**
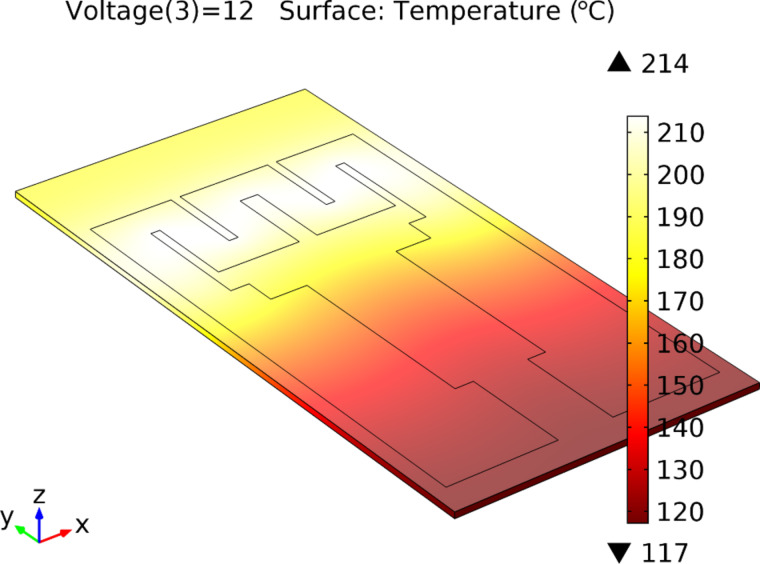
The optimized version of the transducer. The microheater resistive circuit width is 1200 µm (1.2 mm). With an input voltage of 12 V, a maximum achieved temperature of 214 °C is reached.

**Figure 2:**
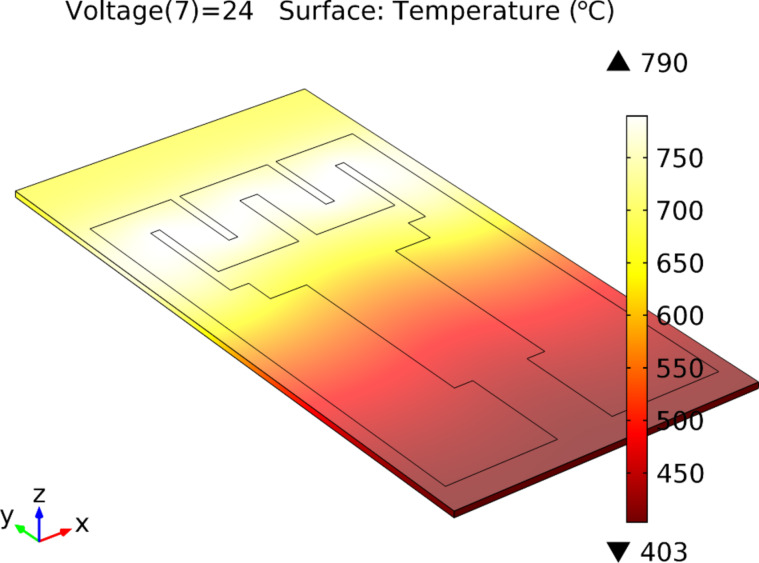
The optimized version of the transducer. The microheater resistive circuit width is 1200 µm (1.2 mm). With an input voltage of 24 V, a maximum achieved temperature of 789 °C is reached.

Up to five versions of the transducer were simulated and fabricated. The final design (fifth version, shown in Figure 1 and Figure 2) provides an excellent temperature range for regular input voltage ranges, quick heating of the substrate (≈20 s) and very good localization of the maximum temperature in the sensor’s active area. The optimized prototype of the transducer is presented in Figure 1 and Figure 2.

**Figure 3:**
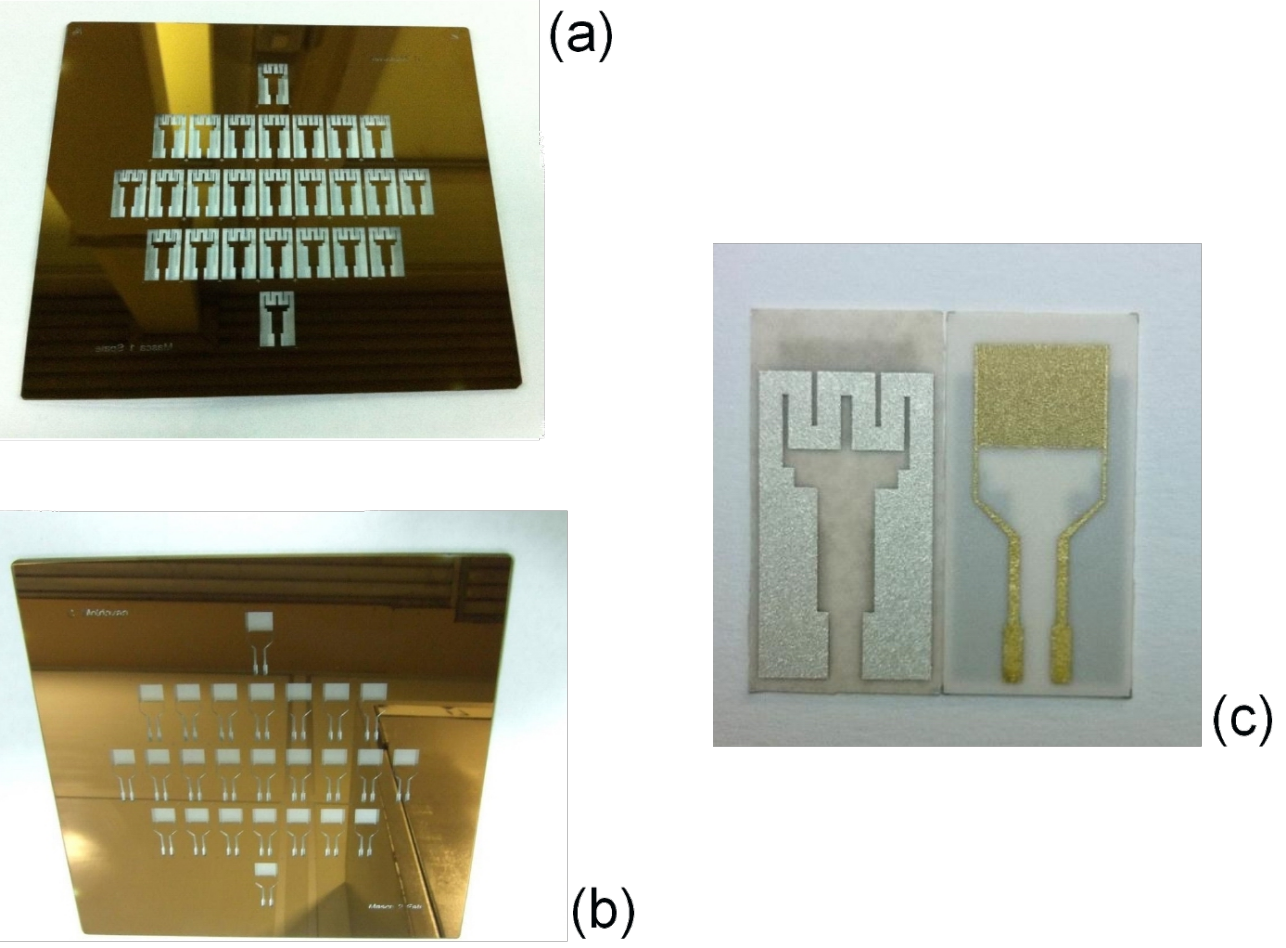
The masks for heater (a), electrode (b) and the fabricated transducer (c).

To verify the obtained simulated results, actual transducers were fabricated and tested. The experimental results were in good agreement with the results obtained in the simulations. Based on these results, the fifth version (see Figure 3) was selected for the deposition of the sensitive layer in order to obtain the final version of the gas sensor.

### Transducer fabrication using mask technology/photolithography

The fabrication of the transducer was carried out using microprocessing technology. The masks for the heater and the interdigital electrode were patterned onto a porous alumina substrate (wafer) using photolithography, etching and lift-off processes.

**Figure 4:**
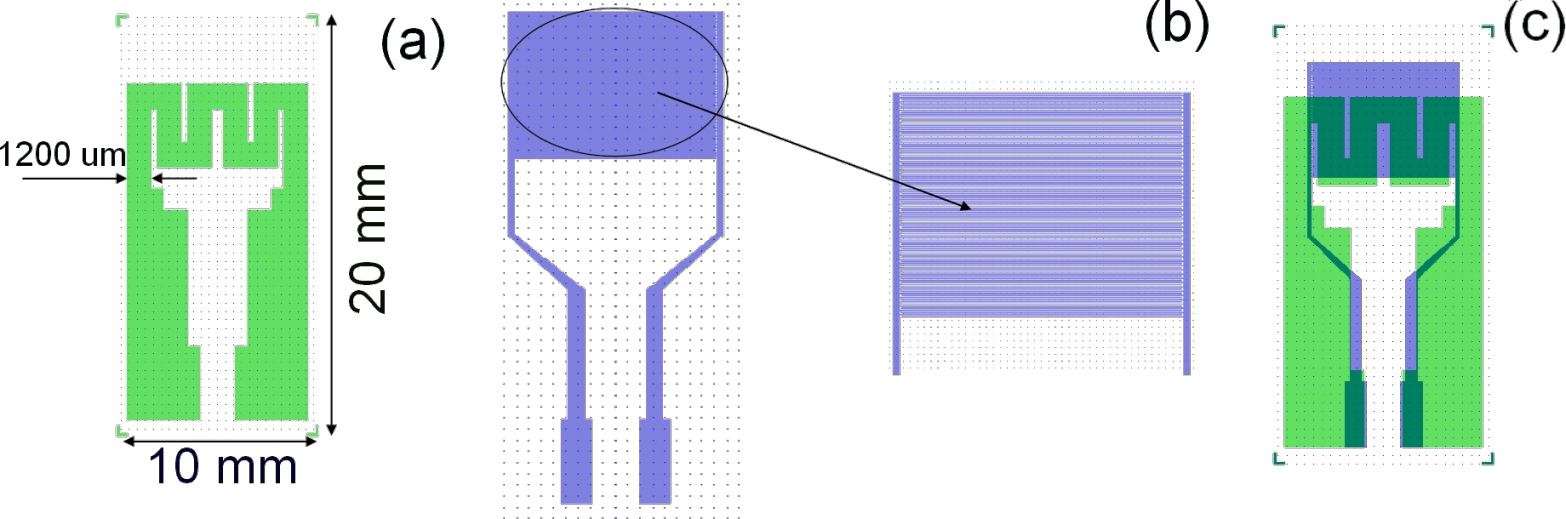
The Pt heater (green) and the Au interdigital electrode (blue) separate (a, b) and superimposed (c) layouts.

Using the working protocol of the laser lithography dedicated equipment (Heidelberg DWL66FS), the design was transferred from the computer to the masks. Using photolithographic process, the structures from the masks were transferred onto the alumina wafer substrate (based on the transducer layout presented in Figure 4), followed by a multistage developing process which uses special reagents and a specific thermal treatment. Figure 4 shows a schematic representation of the heater and the electrode combined to create the final version of the fabricated transducer. The obtained miniaturized transducer has the following dimensions (see Figure 4): 10 × 20 mm (width/length); 200 μm (alumina substrate thickness); 1200 μm (width of the Pt resistor heater). The sensitive area (built on the front side of the alumina wafer) of the transducer has 53 pairs of interdigital electrodes and is 6400 μm in length, 25 μm wide, and the electrode pairs are separated by 25 μm.

### Sensitive layer deposition

The sensitive films were obtained using the low cost, ecologically friendly sol–gel/dip coating method. The precursors, zinc acetate dihydrate (Merck) as a Zn^2+^ source and tin(II) 2-ethylhexanoate (Sigma-Aldrich), were dissolved in ethanol. Triethanolamine (Baker Analyzed) was used as a chelating agent/catalyst. The obtained solutions (0.2 M) were magnetically homogenized for 2 h at 50 °C and left to stabilize for 24 h. The transducers were immersed in the sol for 60 s and then extracted with a speed of 5 mm/s. After each layer deposition, an initial thermal treatment was applied for 10 min at 200 °C. Up to 10 layers were deposited. The final thermal treatment consisted of heating the sensor for 1 h at 350 °C [34]. The obtained sensors were named S_1_–S_5_ (see Table 1).

**Table 1:** The obtained composite sensors and their composition, after final thermal treatment. The sensitive layer deposition was achieved in October, 2015.

Sample	Sensor composition	

	ZnO (wt %)	SnO_2_ (wt %)

S_1_	100	–
S_2_	98	2
S_3_	50	50
S_4_	2	98
S_5_	–	100

### Instrumentation

#### Sensor characterization techniques

The S_1_–S_5_ sensor samples were characterized using scanning electron microscopy (SEM) and atomic force microscopy (AFM). The microstructure of the samples was investigated by SEM using a high-resolution microscope (FEI, Quanta 3D FEG), equipped with an energy dispersive X-ray (EDX) spectrometer (Apollo X). The analyses were performed in high vacuum mode at different accelerating voltages (5–20 kV) and the sensors were analyzed directly (samples were immobilized on a double-sided carbon tape, without coating).

AFM measurements were carried in noncontact mode with an XE-100 apparatus from Park Systems (2011), using sharp tips (<8 nm tip radius; PPP-NCHR type from Nanosensors). The XEI (v.1.8.0) image processing program developed by Park Systems was used for displaying the images and subsequent statistical data analysis.

#### Gas sensing measurements

**Figure 5:**
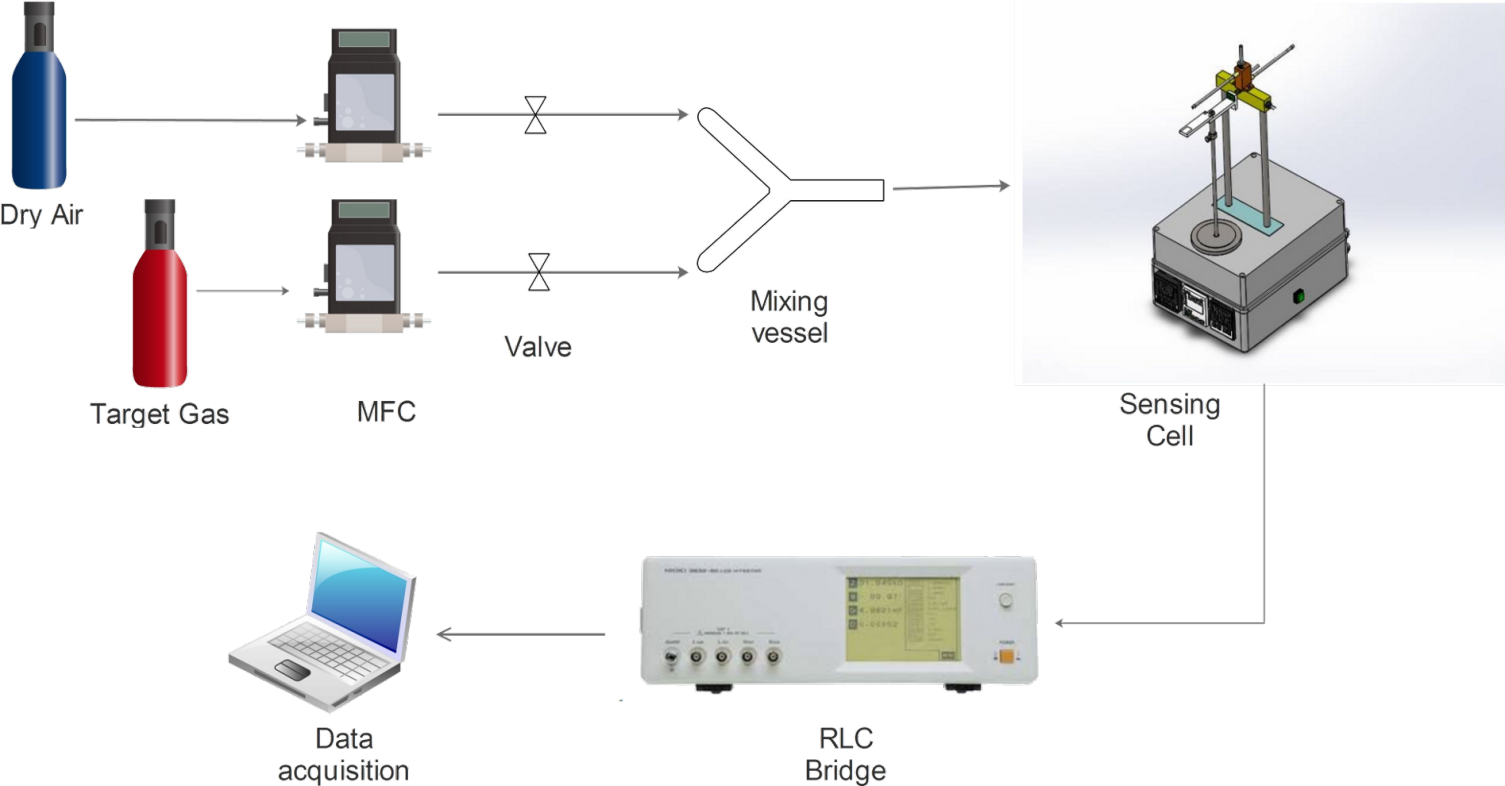
Experimental setup for gas sensing measurements. Adapted from [35], copyright 2016, Elsevier Ltd. and Techna Group S.r.l.

The gas sensing measurements were performed under lab conditions (carrier gas, dry air) using a custom built sensing system (see Figure 5).

**Figure 6:**
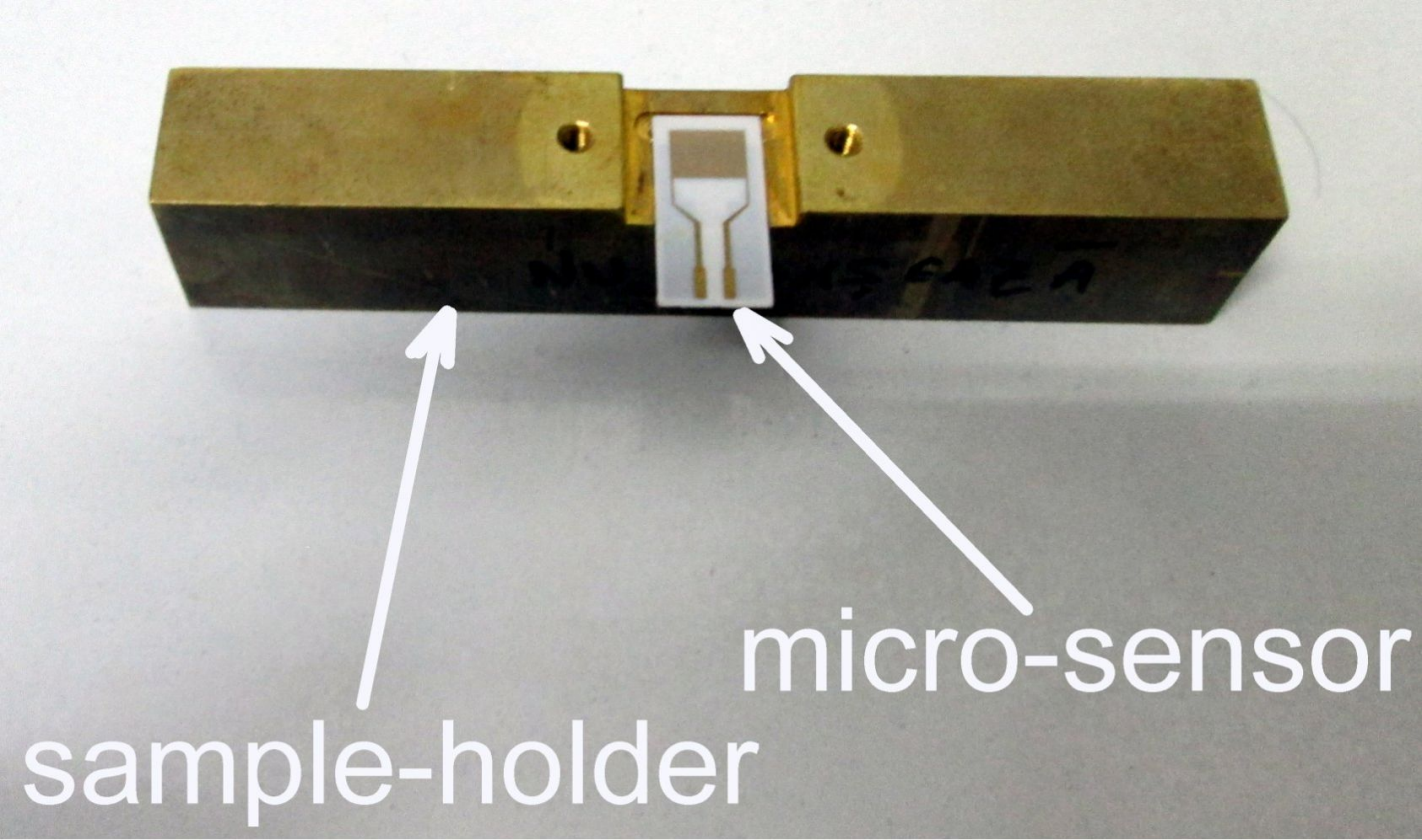
The sample holder/heating platform with the sensor inserted.

The cell includes a heating platform (which is also the sample holder), necessary to reach the working temperature of the sensor (from room temperature to 300 °C, see Figure 6).

**Figure 7:**
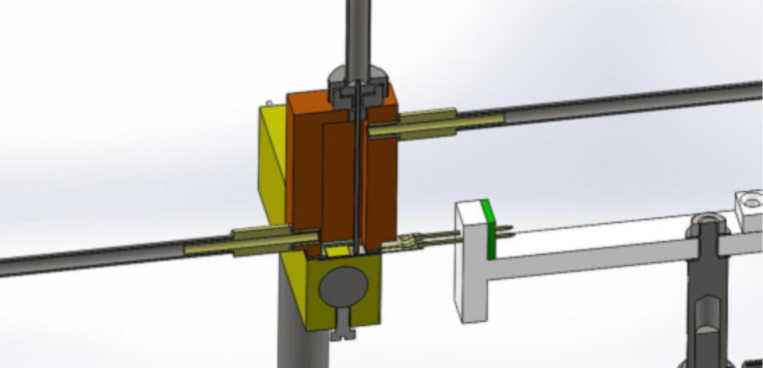
Cross-section of the sensing chamber – left to right: gas inlet, sample holder, thermocouple, gas outlet, and the sensor with the main leads attached.

In the sensing chamber a thermocouple was inserted to give a precise measurement of the working temperature in the atmosphere in which the gas sensor is placed (see Figure 7).

As described in [35], the gas mixing of the carrier and the target gas was performed in a glass vessel placed along the main gas line, before the inlet of the sensing chamber. The resistance of the samples is determined by the atmosphere composition inside the experimental cell. Precise test gas concentrations (5–2000 ppm) are provided by Aalborg mass flow controllers (MFCs). The variation of the electrical resistance was recorded using a Hioki 3522-50 high performance RLC bridge. On the sensor surface, a DC voltage of 1.5 V was applied.

The sensor response was defined as the ratio between the electrical resistance of the sensor in the carrier gas (*R*_air_) and the electrical resistance of the sensor in the target gas (*R*_gas_):

[1]
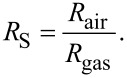


## Results and Discussion

### Sensor morphology

**Figure 8:**
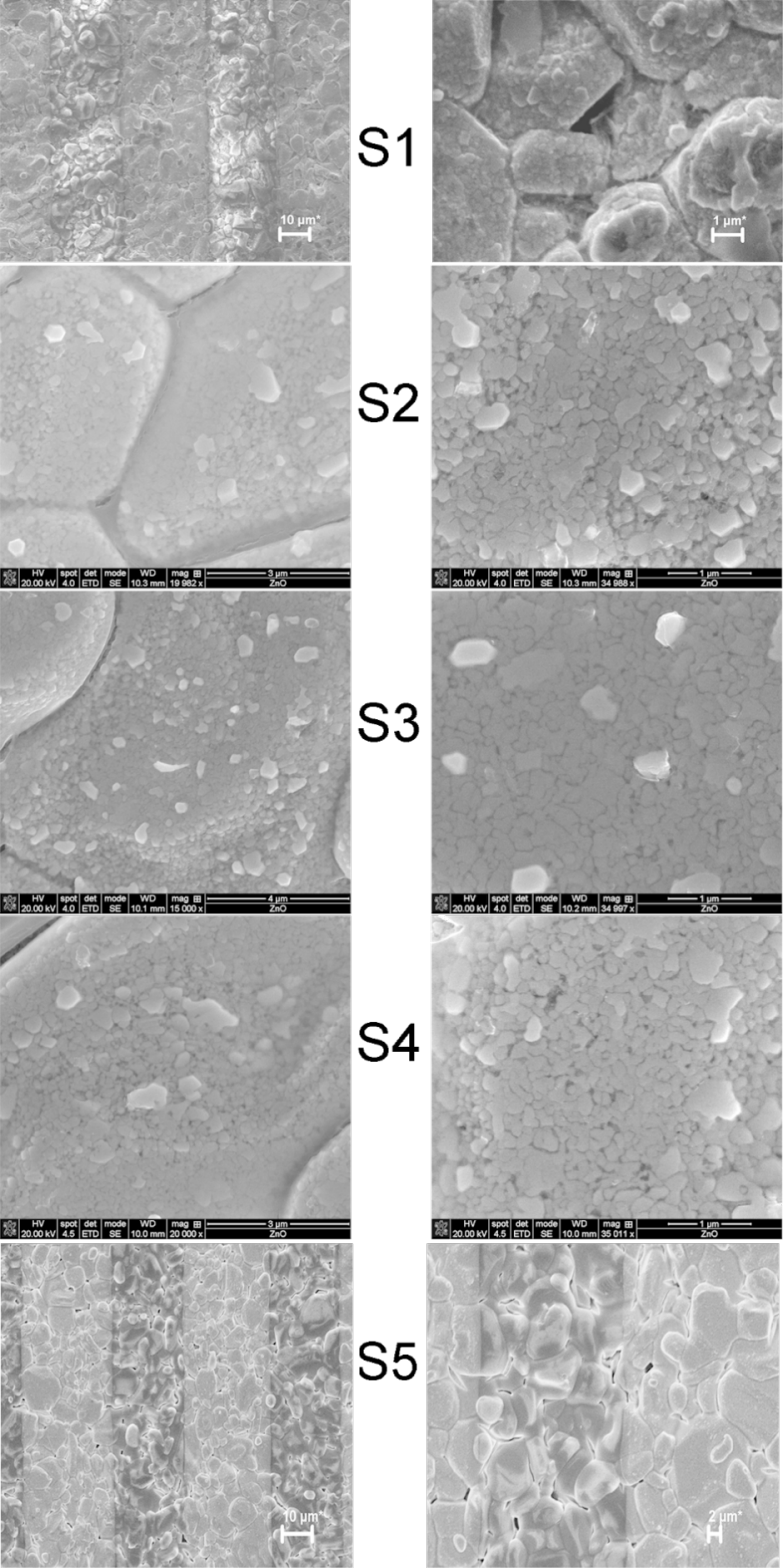
SEM images of the studied sensors (S_1_–S_5_). Gold interdigital electrodes appear as dark grey stripes.

The sensor morphology was investigated by SEM and AFM. From the SEM images (see Figure 8) it can be observed that the thin films deposited onto the miniaturized transducers are continuous (a uniform deposition) and highly transparent. The dark stripes in the SEM images represent the Au interdigital electrodes, which are visible through the films.

Although the films are highly porous, the oxide grains still remain interconnected, and the charge transport mechanism through the oxide film remains unaffected. The films are highly transparent, noticeable as the gold interdigital electrodes are visible through the film. The film grains for the composite samples S_2_, S_3_ and S_4_ were identified with dimensions in the sub-μm range. It can be observed from Figure 8 that the samples with the highest degree of porosity are S_2_ and S_4_. This is a promoting factor for the overall sensing process as more sites for gas adsorption are available for this sample in comparison with the other prepared samples. The large grains observed for the S_1_ and S_5_ samples are actually the alumina grains of the porous alumina wafer. The film structure of the pristine oxides is too compact to obtain high resolution images.

For the ZnO–SnO_2_ composite films, the crystalline structure of the composite samples could not be identified by XRD diffraction measurements, leading to the assumption that the samples exist in amorphous phase. However, the presence of random nanoscale crystallites embedded in an amorphous matrix cannot be ruled out.

**Figure 9:**
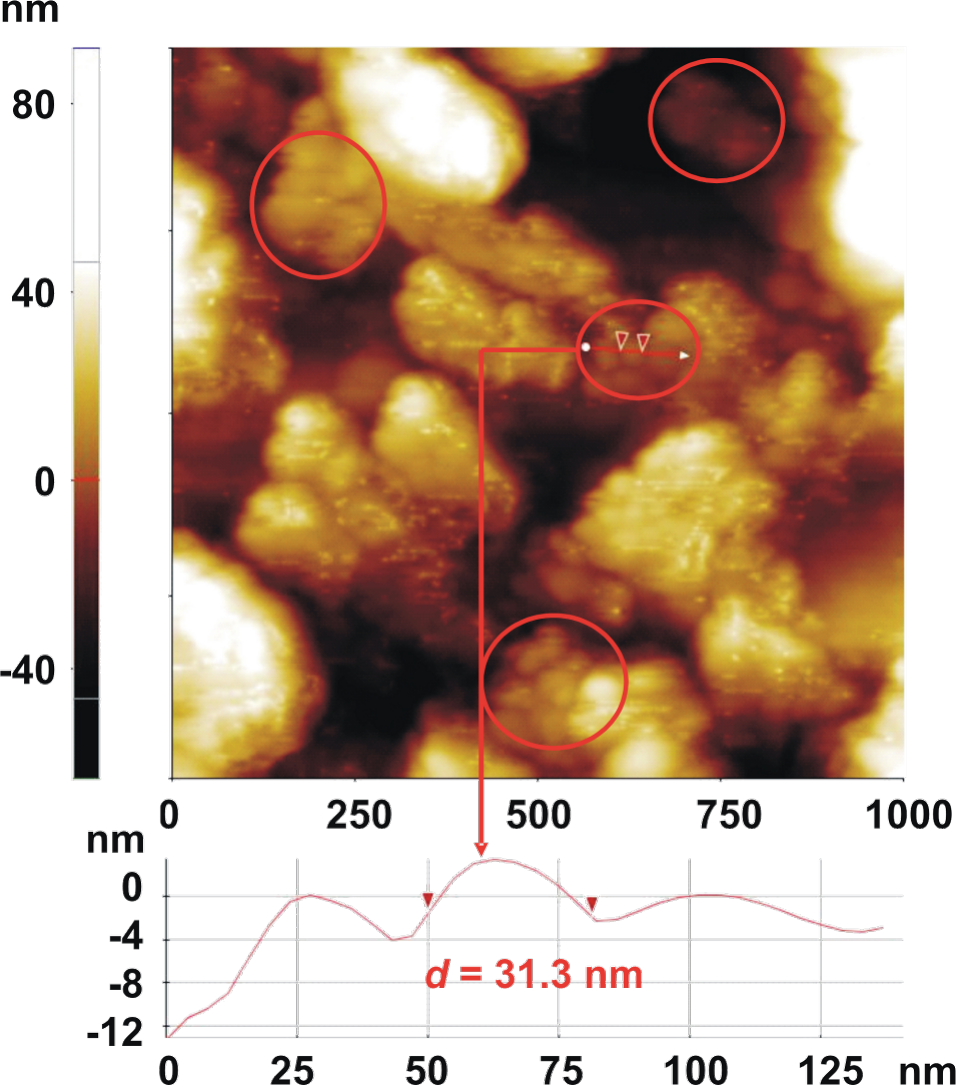
2D AFM image of S_3_ sensor.

AFM measurements were performed at a much higher resolution scale than SEM in order to reveal the morphology (fine grain structure) of the prepared composites. In this sense, the superficial microstructure, consisting in nanometer-sized grains, was identified by AFM (see the areas marked by red circles), exemplified in Figure 9 for the S_3_ sample.

As can be seen, from the selected surface profile (below the AFM image), the typical grain dimension is around 31 nm. Similar values were obtained for all the composite sensors in the prepared series. From the same AFM image, the morphology of the alumina transducer, consisting of large crystallites, could be also observed.

### Gas sensing measurements on the ZnO–SnO_2_ composite sensors

**Figure 10:**
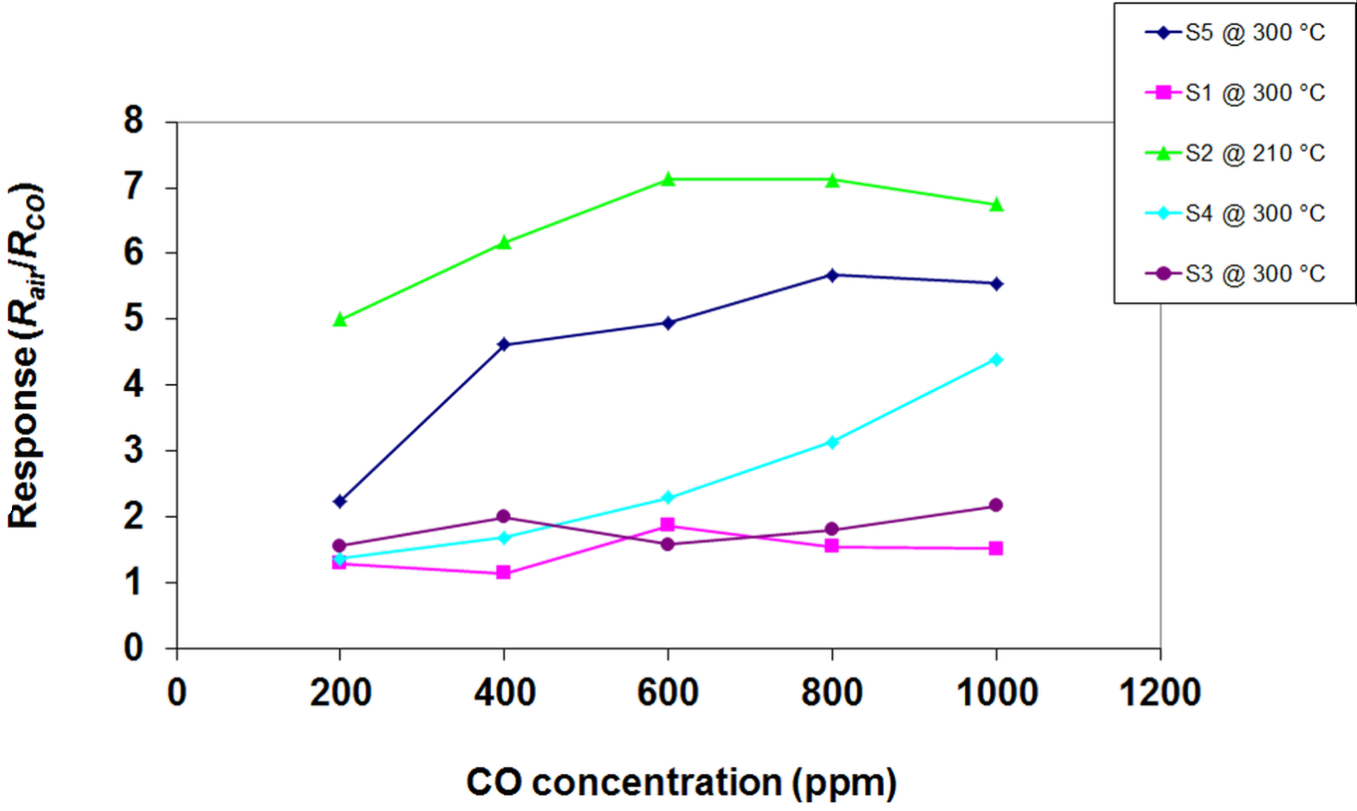
Response of sensors S_1_–S_5_ (recorded in December 2015) to different concentrations of CO at their corresponding working temperatures.

Good sensitivity of ZnO–SnO_2_ composites towards different gases (ethanol, buthanol, dimethyl disulfide, hydrogen, propane, acetone) has been reported in various papers [3,23,27–33], but very few of them have reported selective detection of CO. The response of the prepared composite gas sensors to CO is shown in Figure 10.

**Figure 11:**
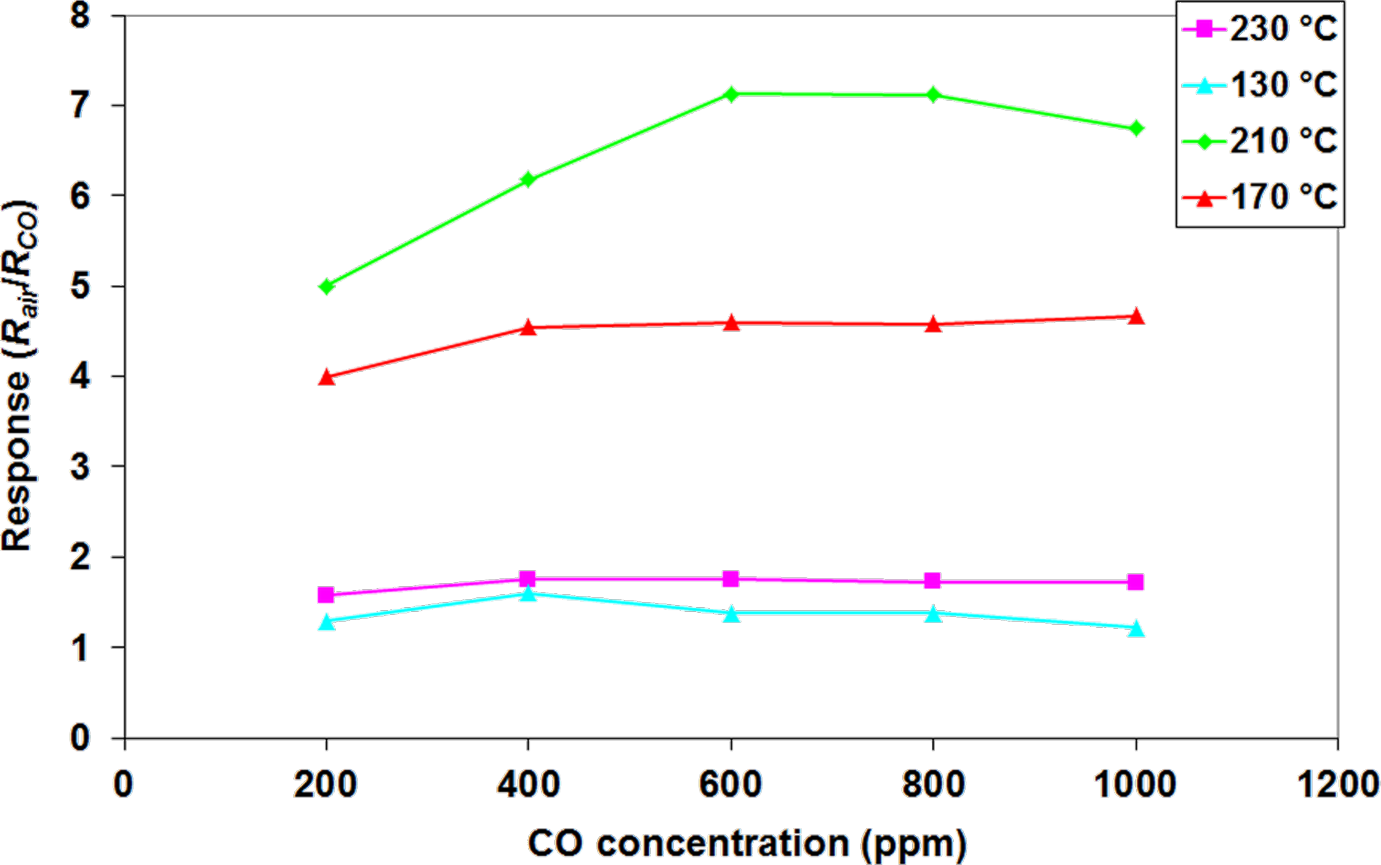
S_2_ response (recorded in December 2015) to CO as a function of the working temperature.

From Figure 10 it is obvious that sensor response to CO is highly influenced by its composition. Pristine ZnO has the lowest response to CO, as expected [36]. With increasing SnO_2_ quantity from 0 to 2 wt % in the S_2_ sample, a high increase in response is observed. A value of *R*_S_ = 7 is obtained for 600 ppm of CO, at a working temperature of 210 °C. This value is higher than the response of the individual components and also higher than all of the other composites. A temperature dependence study is shown in Figure 11 for the S_2_ sensor.

From Figure 11 it can be clearly observed that the optimum working temperature for the S_2_ sensor is 210 °C, and the sensor response at other working temperatures is lower. The second best sensor in terms of CO response is pristine SnO_2_ (S_5_), but later in this paper, it will be shown that its selectivity towards CO is low, as previously reported in literature [36]. The result may be interpreted using the SEM images of the sensitive films, shown in Figure 8. The film with the highest degree of porosity is also the most active film (S_2_) towards CO detection.

An additional study was previously published by the authors of this paper where tin–zinc ceramic composites were investigated [35]. In this study the conductivity changes leading to different sensor response were motivated by the presence of different phases having different electric behaviors (ZnO, SnO_2_, and Zn_2_SnO_4_ phases were identified in the composite sensing materials, using XRD). The identification of these phases was not possible in the present case as the thin films were amorphous, but as mentioned before, the presence of the crystallites in an amorphous matrix was not ruled out.

### Sensor response to humidity

**Figure 12:**
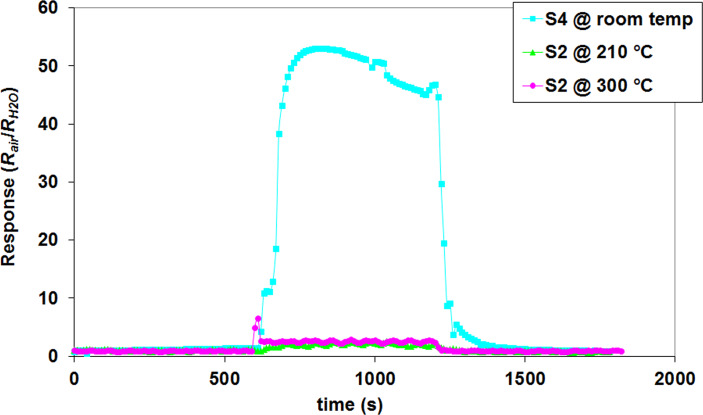
Different composite sensor responses to relative humidity (62%).

In real life applications, environmental humidity is an important factor which influences sensor response. In Figure 12 different sensors were tested in an atmosphere containing 62% relative humidity.

It can be observed that the sensor having the highest response to CO (S_2_) is only slightly influenced by humidity at its optimum working temperature. The response to water vapor is very low when compared to the S_4_ composite sensor which has a very high response to humidity even at room temperature. This sensor is being considered for the further development of a humidity sensor.

### Sensor cross-response measurements

To avoid false positives, gas sensors must be selective towards a specific gas in a given gaseous environment. This is still a challenging issue for the commercially available gas sensors.

As it was discussed in the introduction section of this article, selectivity towards a specific gas may be tuned by using composite materials. For the prepared series, the cross-response to CO, CO_2_, CH_4_ and C_3_H_8_ was measured. Each sensor was exposed separately to a specific concentration and the corresponding sensor response was automatically recorded.

**Figure 13:**
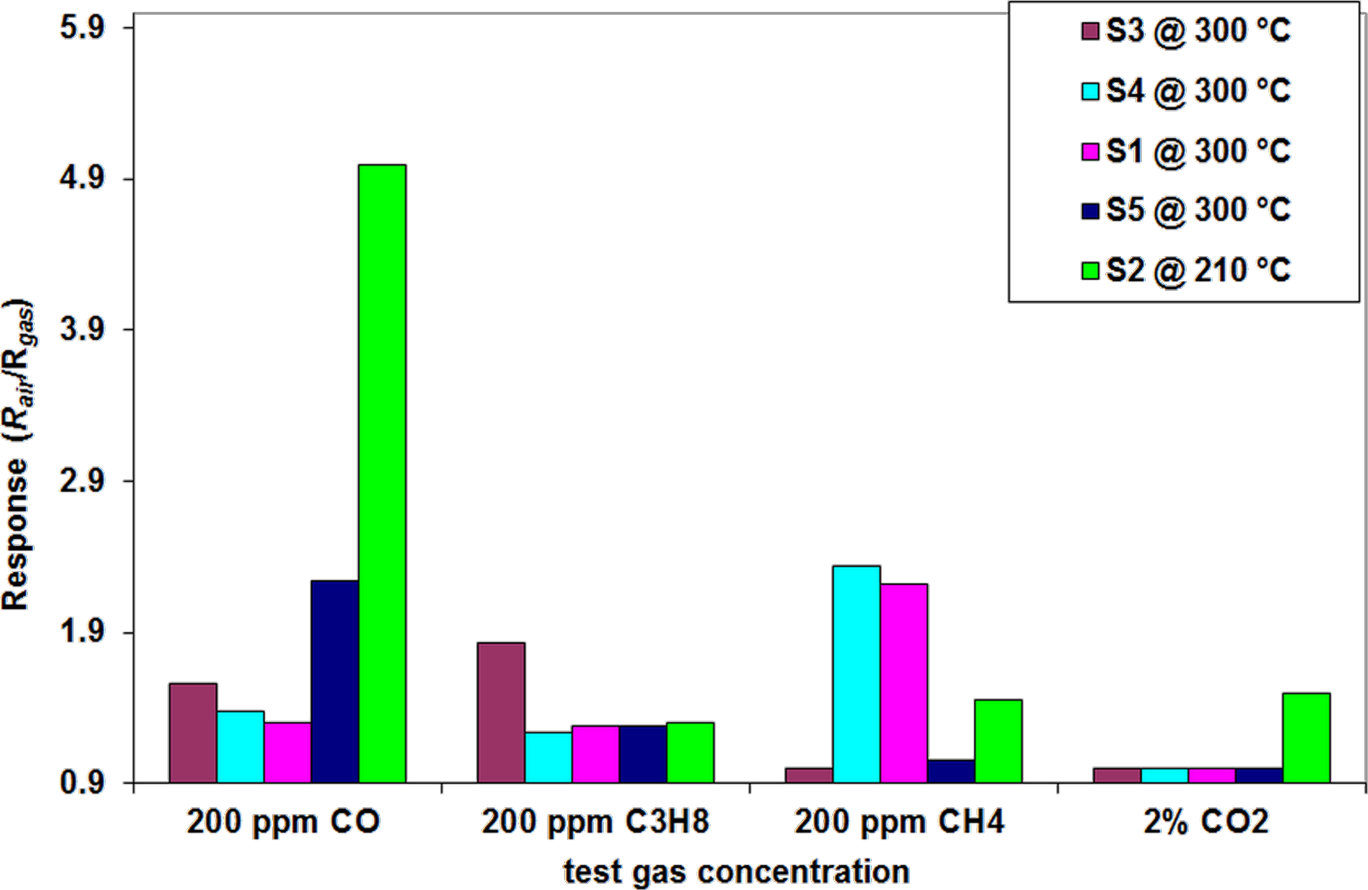
Sensor cross-response (recorded in December 2015) to different concentrations of gases, at the corresponding optimum working temperature of the sensor.

From Figure 13 it can be observed that the sensor with the best response and the highest selectivity towards CO is S_2_, at a corresponding optimum working temperature of 210 °C. Its response is approximately 5× higher compared to its response towards CO_2_, methane and propane. According to these results it can be said that the sensor has a good selectivity to CO.

It can be seen from Figure 13 that the S_5_ sample (pristine SnO_2_) has comparable response for all the tested gases; it is considered to be sensitive to these gases but nonselective towards CO.

### Sensor response–recovery characteristics

**Figure 14:**
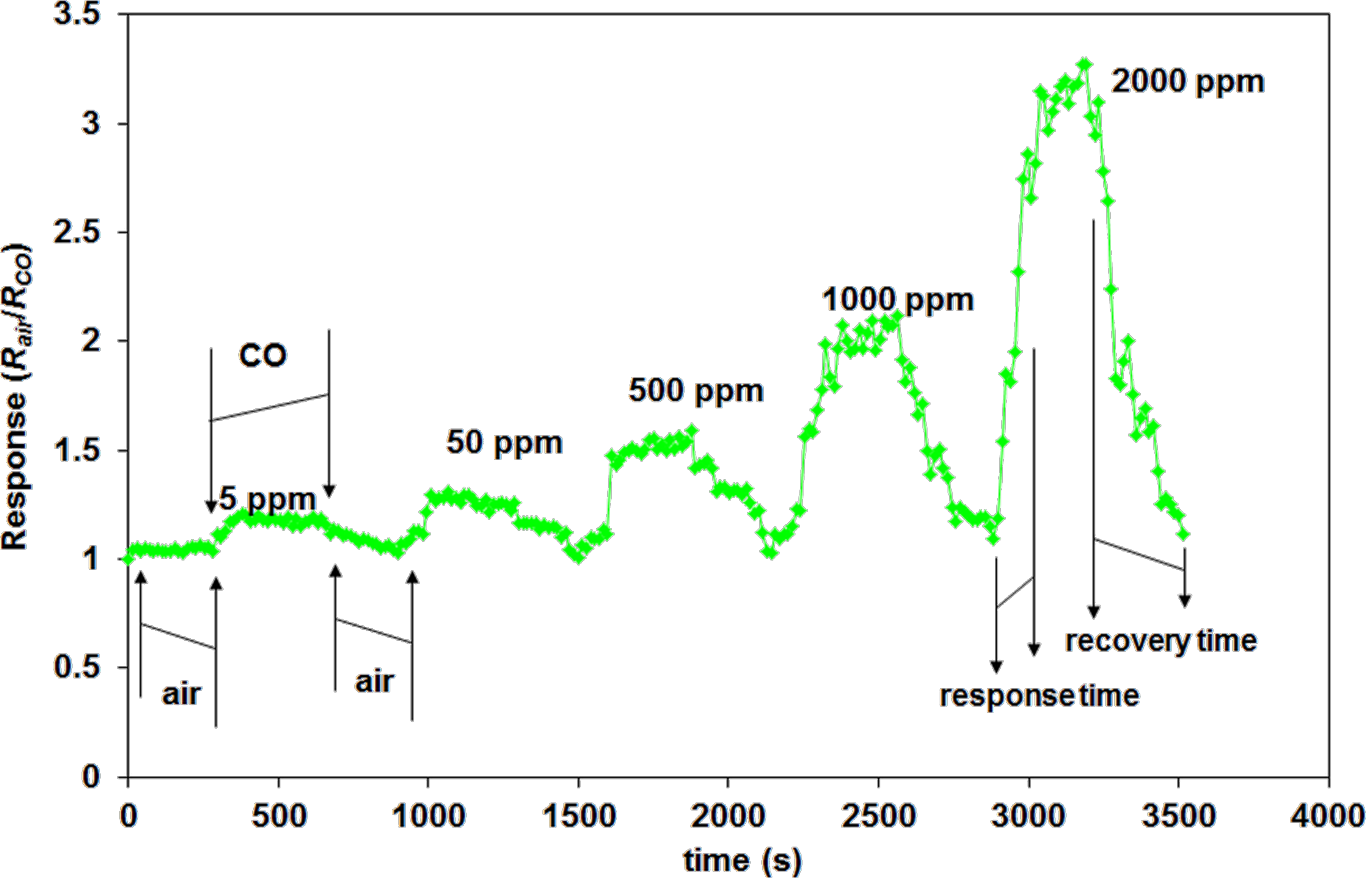
Sensor response and recovery characteristics (recorded in June 2016) for sensor S_2_ at 300 °C, for different CO concentrations.

Sensor recovery characteristics were recorded for all the obtained sensors in the 200–1000 ppm range (see Figure 10) and also in the wider range of 5–2000 ppm for the best sensor in the prepared series (S_2_), as shown in Figure 14. The measurements were made after leaving the sensor unprotected in the atmospheric environment for six months to test changes in the sensing performance and stability. The other sensors were nonsensitive in the low concentration domain (5–200 ppm).

It can be seen from Figure 14 that the sensing performance is diminished as compared to the values obtained in Figure 10. However, the sensor is stable during the measurements for all the tested concentrations, and good sensitivity to CO is still obtained. The good response time of 120 s and a complete sensor recovery was achieved in 190–280 s for the S_2_ sensor (see Figure 9), at a working temperature of 300 °C. The response value returns to the baseline after each tested concentration if the sensitive coating is decontaminated by heating the sensor in the carrier gas for a period equal (at least) to the recovery time.

**Figure 15:**
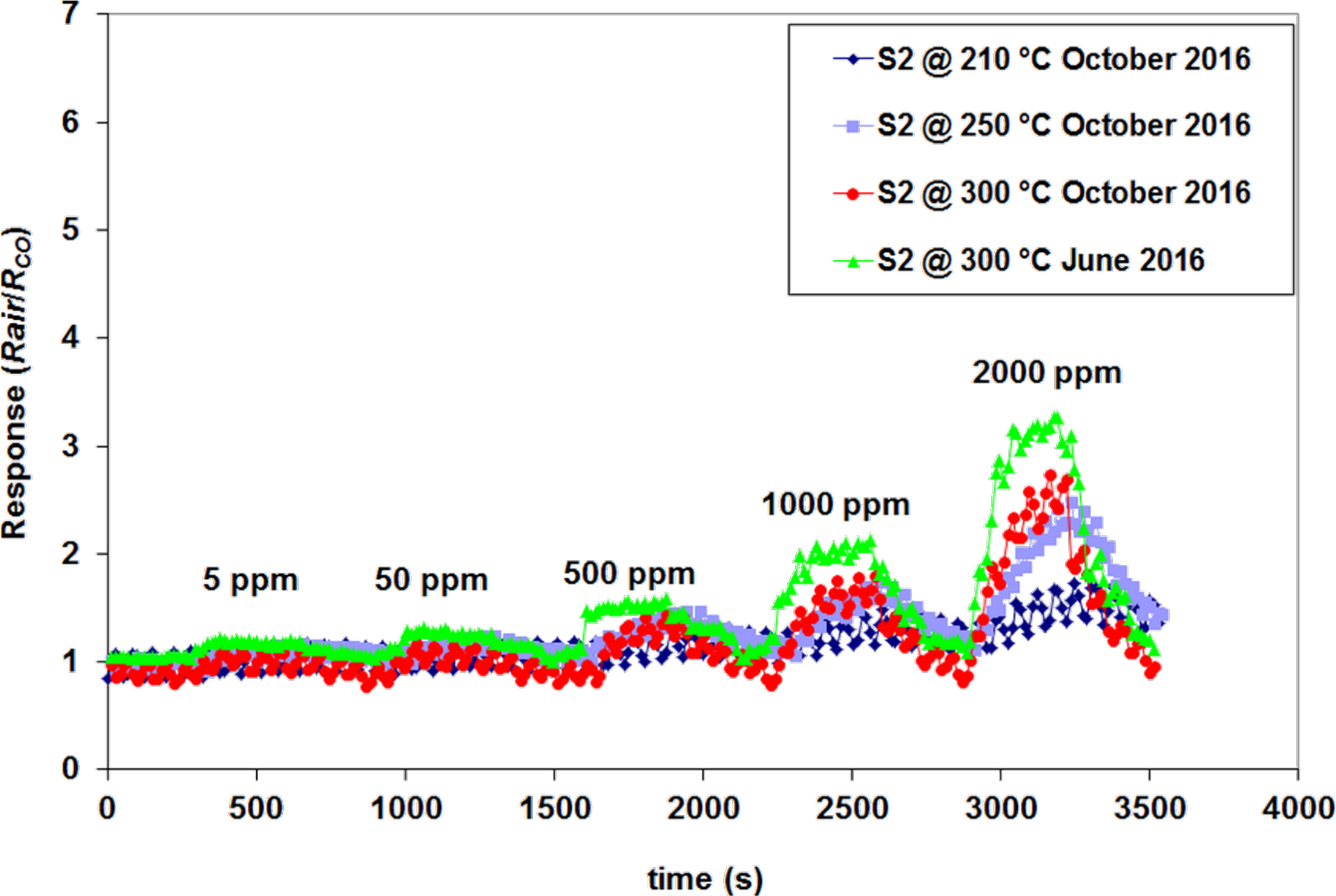
S_2_ response for different working temperatures.

The S_2_ sensor was found to be sensitive even to low concentrations of CO (5 ppm) as seen in Figure 14, with a response value of 1.21. Other working temperatures were tested for the S_2_ sensor to verify if the sensor performance really diminishes over time. The response curves are shown in Figure 15.

**Figure 16:**
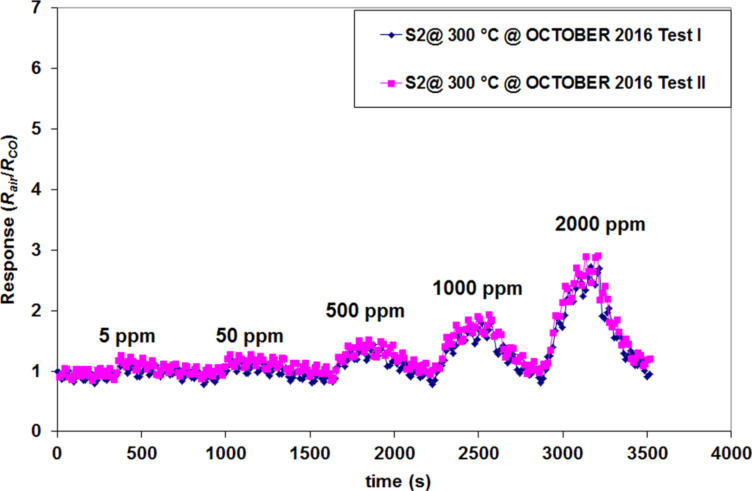
S_2_ sensor response for different tested CO concentrations, under identical test conditions.

From Figure 15 it appears that the sensor performance diminishes over time. A very low response is recorded for the initial optimum working temperature of 210 °C. After one year from the deposition of the sensitive layer, the optimum working temperature has changed from 210 to 300 °C. This is an inherent feature of the thin films, which degrade and lose their sensing performance over time. The film stability in the present case is considered to be fair, giving the fact that the first sensing results were recorded in December 2015 (with the highest response values, see Figure 10), and the final measurements were performed during October 2016, with good sensing results even at low CO concentrations. It can also be observed that with decreasing working temperature from 300 to 210 °C the noise in the sensor response signal increases. Multiple tests were performed using the same working protocol to test the actual response reproducibility. The results are shown in Figure 16.

It can be clearly observed that under the same test conditions the S_2_ sensor yields an identical response for each testing cycle. The sensor response value returns to the baseline after each tested concentration, as can be observed in Figures 14–16. The results are thus considered reproducible.

## Conclusion

ZnO–SnO_2_ composite nanostructured sensors with different SnO_2_ content were prepared via an ecologically friendly, low cost technique (sol–gel/dip coating) in order to obtain an improved sensor in terms of selectivity towards CO. The transducers and the gas sensing measurement cell were custom built.

All the studied sensors presented similar morphology having amorphous structure and a high transparency of the sensitive films. One particular sample (S_2_, containing 2% SnO_2_ and 98% ZnO) had a very high porosity – a feature which promotes the gas adsorption on the surface sites, improving the overall sensing properties of the studied material.

The response of the obtained sensors was tested by exposure to different gases. The sensor response increases for the composite sensors as compared to the sensors which contain only the pristine oxides. It was found that the S_2_ sensor has the highest response, up to five times higher, as compared with the response of the pristine ZnO sensor and also has a good selectivity towards CO. The S_2_ electrical response was five times lower for C_3_H_8_, CH_4_ and CO_2_ at an optimum working temperature of 210 °C.

The sensor S_2_ response was averaged at 120 s with good recovery characteristics (complete recovery at a maximum of 280 s). One year after deposition of the sensitive coating (October 2015–October 2016) the best sensor in the prepared series (S_2_) was found to be sensitive even to low concentrations of CO (5 ppm), at a working temperature of 300 °C. The sensitive film had fair stability over time, but noting that the sensor response to CO slowly diminishes over time and the optimum working temperature of the sensor increases from 210 to 300 °C. Given that the sensing results are reproducible, the sensor may be proposed for further development which may result in a commercial sensor for selective CO detection.

The composite sensor, S_4_, containing 2 wt % ZnO and 98 wt % SnO_2_ exhibited a very high response to humidity at room temperature. This sensor may be proposed for further development of a humidity sensor.
